# A novel biosafety level 2 compliant tuberculosis infection model using a Δ*leuD*Δ*panCD* double auxotroph of *Mycobacterium tuberculosis* H37Rv and *Galleria mellonella*

**DOI:** 10.1080/21505594.2020.1781486

**Published:** 2020-06-24

**Authors:** Masanori Asai, Yanwen Li, John Spiropoulos, William Cooley, David Everest, Brian D. Robertson, Paul R. Langford, Sandra M. Newton

**Affiliations:** a Section of Paediatric Infectious Disease, Department of Infectious Disease, Imperial College London, London, UK; b Department of Pathology, Animal and Plant Health Agency, Addlestone, UK; c MRC Centre for Molecular Bacteriology and Infection, Department of Infectious Disease, Imperial College London, London, UK

**Keywords:** *Galleria mellonella*, tuberculosis, infection model, *Mycobacterium tuberculosis* complex, drug screening, antimycobacterial agents, mycobacteria, auxotrophic

## Abstract

Mammalian infection models have contributed significantly to our understanding of the host-mycobacterial interaction, revealing potential mechanisms and targets for novel antimycobacterial therapeutics. However, the use of conventional mammalian models such as mice, are typically expensive, high maintenance, require specialized animal housing, and are ethically regulated. Furthermore, research using *Mycobacterium tuberculosis* (MTB), is inherently difficult as work needs to be carried out at biosafety level 3 (BSL3). The insect larvae of *Galleria mellonella* (greater wax moth), have become increasingly popular as an infection model, and we previously demonstrated its potential as a mycobacterial infection model using *Mycobacterium bovis* BCG. Here we present a novel BSL2 complaint MTB infection model using *G. mellonella* in combination with a bioluminescent *ΔleuDΔpanCD* double auxotrophic mutant of MTB H37Rv (SAMTB *lux*) which offers safety and practical advantages over working with wild type MTB. Our results show a SAMTB *lux* dose dependent survival of *G. mellonella* larvae and demonstrate proliferation and persistence of SAMTB *lux* bioluminescence over a 1 week infection time course. Histopathological analysis of *G. mellonella*, highlight the formation of early granuloma-like structures which matured over time. We additionally demonstrate the drug efficacy of first (isoniazid, rifampicin, and ethambutol) and second line (moxifloxacin) antimycobacterial drugs. Our findings demonstrate the broad potential of this insect model to study MTB infection under BSL2 conditions. We anticipate that the successful adaptation and implementation of this model will remove the inherent limitations of MTB research at BSL3 and increase tuberculosis research output.

## Introduction

*Mycobacterium tuberculosis* (MTB) is the causative agent of tuberculosis (TB), the leading cause of global infectious disease mortality. In 2018, there were 10 million cases and 1.6 million deaths from TB [1]. One quarter of the world’s population have latent TB infection (LTBI), of which 10% will go on to develop active disease over their lifetime [1]. Treatment of TB is challenging, especially with the rise of drug resistant TB and the lack of efficacious treatments [2]. Only three new drugs have been approved by the US Food and Drug Administration (FDA) in almost five decades [3,4]. Furthermore, preventative measures are limited with only one FDA approved vaccine, Bacillus Calmette-Guérin (BCG), which has variable efficacy [5]. The World Health Organization (WHO) target to end the global TB epidemic by 2035 [1], which is a highly improbable goal taking into account current progress [2]. In order to end the global TB epidemic, significant advancement in research is needed to develop novel treatments, preventative measures and diagnostic tools.

Animal infection models have played a substantial role in TB research leading to an increased understanding of host-bacterial interactive biology, and are crucial for novel therapeutic and vaccine development [6–8]. The mouse, rabbit, guinea pig, non-human primate and zebrafish (*Danio rerio*) have all been used [6]. However, each model has limitations with no single model being able to replicate all aspects of the disease. For example, the widely used mouse strains (C57BL/6 and BALB/c) do not develop necrotic granulomas, a hallmark phenotype of LTBI [9]. Rabbits and guinea pigs do develop human-like necrotic granulomas, but the lack of immunological reagents limits their use [6]. Most mammalian models are not naturally susceptible to mycobacteria and are time consuming. Additionally, such models require specialized biological containment (biological safety level 3, BSL3), are expensive, and require ethical approval. These limitations highlight the need for alternative infection models that can accelerate our understanding of TB biology and the development of novel therapeutics.

Over the past decade, the insect larva of *Galleria mellonella* (greater wax moth) has become a popular model to study the pathogenesis of Gram-negative and Gram-positive bacteria, and fungal pathogens [10,11]. Additionally, the model has been used for rapid drug efficacy testing [10,12,13]. The popularity of *G. mellonella* stems from its complex innate immune system, which shares high structural and functional similarity to that in mammals. *G. mellonella* has many advantages over conventional models: 1) larval maintenance does not require specialized housing: 2) last instar larvae do not need feeding: 3) no specialized equipment is required: 4) their size (2–3 cm) permits easy handling and precise dosing of pathogen and/or drugs: 5) they can be incubated at 37°C (optimum temperature for human pathogens); and 6) ethics permission is not required [14]. In previous studies using *Mycobacterium bovis* BCG as a surrogate for MTB, we demonstrated that *G. mellonella* can be used to study members of the MTB complex (MTBC) [14,15], including screening of antimycobacterial agents [14].

A recent publication described a BSL2 compliant, severely attenuated double auxotrophic ∆*leuC*∆*panCD* strain of MTB H37Rv (SAMTB) [16]. Compared to H37Rv (wild-type, WT), SAMTB had comparable *in vitro* and intracellular growth, and immunogenicity, making it a suitable surrogate for WT MTB [16]. In this study, we have characterized a novel BSL2 compliant SAMTB-*G. mellonella* infection model, and show its potential to accelerate TB research.

## Materials and methods

### Bacterial growth conditions

The double auxotrophic *M. tuberculosis* H37Rv Δ*leuD* Δ*panCD* mutant (SAMTB) was kindly provided by Professor William Jacobs Jr. of Albert Einstein College of Medicine. SAMTB was transformed into an auto-luminescent mutant (SAMTB *lux*), using the plasmid vector pMV306, carrying the full *lux* operon of *Photorhabdus luminescens* [17]. The use of pMV306 was chosen for its stability, as the plasmid is chromosomally integrated. The resistance status of SAMTB *lux* is similar to that of WT *M. tuberculosis* H37Rv [16], with additional resistance to kanamycin and hygromycin for Δ*leuD*Δ*panCD* and pMV306 selection respectively. SAMTB *lux* was cultured in Middlebrook 7H9 broth (BD, UK), supplemented with glycerol (0.2%) [Sigma-Aldrich, UK], albumin dextrose catalase (ADC 10%) [USBio, USA], polysorbate-80 (0.05%) [Sigma-Aldrich, UK], leucine (25 µg/ml) [Sigma-Aldrich, UK) and calcium pantothenate (24 µg/ml) [Sigma-Aldrich, UK]. The broth was further supplemented with hygromycin (50 µg/ml) and kanamycin (20 µg/ml) for pMV306 selection. For enumeration of colony forming unit (CFU)/ml, SAMTB *lux* was serially diluted ten-fold and each dilution was plated onto 7H11 agar (BD Difco, UK) supplemented with oleic albumin dextrose catalase (OADC 10%) [BD, UK], glycerol (0.5%), leucine (25 µg/ml) and calcium pantothenate (24 µg/ml), hygromycin (50 µg/ml) and kanamycin (20 µg/ml). SAMTB *lux* cultures were grown at 37°C with agitation (220 rpm) to mid-log phase and CFU enumeration was determined on agar plates incubated at 37°C with 5% CO_2_ for 2 weeks. Growth of SAMTB *lux* was monitored through measurements of turbidity (OD 600 nm) and bioluminescence (relative light units, RLU) using a luminometer (Berthold Technologies, DE), where the RLU:CFU ratio had been determined to be 0.03:1 (Supplementary Figure 1). This ratio was maintained throughout the 96 h incubation, with the exception to 24 h, likely as a result of metabolic adjustment during the lag phase of growth.

**Figure 1.**
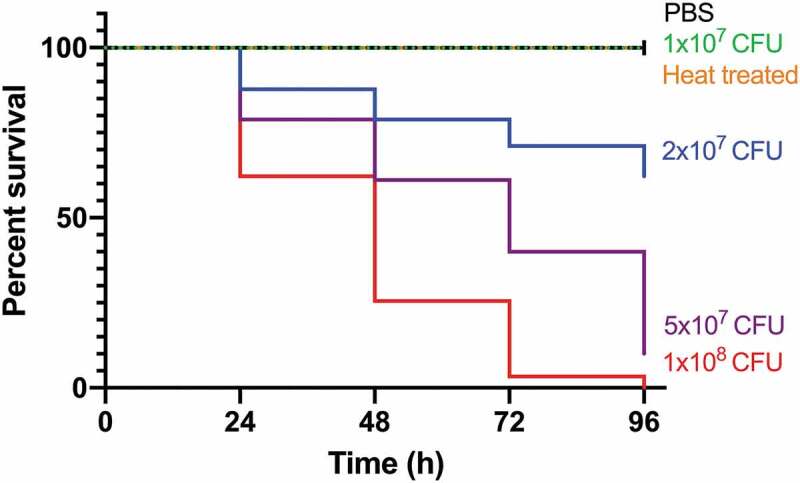
Kaplan-Meier survival curves of *G. mellonella* challenged with a range of SAMTB *lux* inocula over a 96 h time course.

*G. mellonella* larvae (n = 30/group) were infected with 1 × 10^7^–10^8^ CFU of SAMTB *lux*. Infected larvae were stored in a dark box and incubated at 37°C. Survival was recorded every 24 h for a period of 96 h. Larvae were considered dead when they failed to move in response to touch. All SAMTB *lux* doses were statistically different from each other (P < 0.0001, Mantle-Cox test with Holm-Sidak correction) at each timepoint. Data were pooled from three independent experiments.

### G. mellonella *maintenance*

Last-instar *G. mellonella* larvae were purchased from Livefoods Direct Ltd, UK. Healthy larvae were defined by their uniform cream color, lack of melanization, body length (2–3 cm), weight (~250 mg), and high level of motility with the ability to right themselves when turned over. Dead, melanized or pupating larvae were discarded prior to storage at 18°C. Larvae were not fed prior to storage or during infection. Larvae were stored for no longer than 1 week before experimentation.

### *Preparation of mycobacterial inocula and infection of* G. mellonella

All experiments with SAMTB were performed in a BSL2 laboratory. Mid-log phase SAMTB *lux* culture was pelleted for 10 min at 2175 g at room temperature. The cell pellet was washed twice with phosphate buffered saline (PBS) [Sigma-Aldrich, UK], containing 0.05% polysorbate-80 (PBS-T). Following the final wash, the cell pellet was resuspended to the desired inoculum density using RLU and OD measurements, with the CFU being enumerated for each inoculum on agar plates. Healthy larvae were selected and infected with SAMTB *lux* as previously described [14]. Briefly, each larva was injected with a 10 µl dose of SAMTB *lux* (2x10^7^ CFU) using a micro-syringe (SGE Analytical Science, AU), via the last left pro-leg directly into the hemocoel. Infected larvae were counted into a Petri dish (94 mm), lined with a layer of filter paper (n = 30, per group/Petri dish), and incubated in a vented box at 37°C in the dark. Heated treated inocula comprised, 2×10^7^ CFU heated at 85°C for 1 h.

### *Treatment of infected* G. mellonella *using antimycobacterial compounds*

All antibiotics were purchased from Sigma-Aldrich, UK and prepared following the manufacturer’s guidelines. Isoniazid (INH, 5 mg/kg), rifampicin (RIF, 10 mg/kg), ethambutol (ETH, 15 mg/kg), pyrazinamide (PZA, 25 mg/kg), moxifloxacin (MOX, 6.7 mg/kg) and bedaquiline (BDQ, 5.7 mg/kg) were used at recommended clinical dosages for adults following WHO guidelines [18], relative to the body weight of the larvae. Larvae were subjected to mono, dual (RIF/ETH) or triple (RIF/ETH/MOX) drug combination treatment in a single or double injection schedule. Treatment was carried out as previously described [13]. Briefly, larvae were treated 1 h post-infection via injection into the last left pro-leg; and for multiple dosing, a second dose was injected 24 h post-infection.

### Minimum inhibitory concentration (MIC) assay

Mid-log phase SAMTB *lux* culture was diluted to 10^6^ CFU/ml in fresh 7H9 broth supplemented with leucine and pantothenate. Kanamycin and hygromycin were omitted as they are non-essential for growth. All drugs were diluted in 7H9 to provide serial 1:1 dilutions ranging from 16 µg/ml to 0.125 µg/ml. One hundred microliters of SAMTB *lux* was seeded into the wells of a 96 well plate in triplicates. A further 100 µl of the drug dilutions were added to these wells in triplicates. Positive controls were SAMTB *lux* alone and negative controls were broth only. Only the inner wells of the plate were used for the assay to prevent evaporation; the outer ring of wells were filled with water to reduce the edge effect. The lidded plates were transferred to an airtight container lined with moistened paper towels to minimize evaporation. The plates were incubated at 37°C with agitation (20 rpm) for 1 week and the OD of the wells (520 nm) were read in a plate reader (Molecular Devices, UK). The MIC, defined as the minimum concentration of antibiotics required to inhibit the growth of SAMTB *lux*, was determined using two independent experiments.

### In vivo *virulence assay of SAMTB* lux *in* G. mellonella

Larvae (n = 30 per group) were challenged with varying SAMTB *lux* inocula. Larval survival was recorded every 24 h, over a 96 h time course. Larvae were considered dead when they failed to respond to touch. Larvae which were pricked (to mimic needle injury) or PBS injected, were used as controls. Survival of larvae were plotted as a Kaplan-Meier survival curve from a minimum of two experimental repeats. Pupae were removed from the sample groups and omitted from the analysis.

### *Measurement of* in vivo *survival of SAMTB* lux *in* G. mellonella *and relative drug efficacy*

Larvae were infected with 2×10^7^ CFU of SAMTB *lux* and relative changes in the *in vivo* mycobacterial loads were measured using bioluminescence over a one week time course. At each timepoint (0, 24, 48, 76, 92 and 168 h), five larvae were randomly selected and individually homogenized with 800 µl of PBS-T in a 2 ml lysis tube with six 1/8 inch metal beads (MP Biomedicals, US), using the FastPrep-24 5 G instrument (MP Biomedicals, US). Samples were homogenized at machine setting 6.0 m/s for 1 minute. The bioluminescence of the homogenate was measured using a luminometer. The survival of SAMTB *lux in vivo* was determined using data pooled from a minimum of three experimental repeats. The homogenates were plated onto 7H11 agar to enumerate the CFU to determine RLU:CFU ratio *in vivo*: 7H11 agar was further supplemented with piperacillin (20 µg/ml) to inhibit the growth of *G. mellonella* flora as previously described [15].

### Total hemocyte count (THC)

Larvae (n = 5, per group) were bled through puncture of the last right pro leg using a sterile 30 gauge needle (Terumo, Japan) and three drops of hemolymph (approximately 60 µl) were collected into an ice cold 1.5 ml Eppendorf tube. Hemolymph from each larva were pooled (40 µl/larva, total 200 µl) and diluted with ice cold PBS containing 0.37% of mercaptoethanol (Sigma-Aldrich, UK)). Diluted hemolymph was stained 1:1 with 0.4% trypan blue (Thermo Fisher, UK), and hemocytes counted using an automated hemocytometer (Thermo Fisher, UK). Bleeding of larvae is a terminal procedure. Larvae were bled at 2, 4, 24, 48 h post-infection. Data were pooled from three independent repeats, each with two technical repeats.

### *Histopathological, confocal microscopy and transmission electron microscopy (TEM) analysis of SAMTB* lux *infection in* G. mellonella

*G. mellonella* larvae were infected with SAMTB *lux* (2x10^7^ CFU) for histopathological and TEM time course analysis, performed as previously described [15]. For histopathology, three larvae were fixed and stored in buffered formalin at 0, 24, 48 and 168 h post-infection. Uninfected healthy larvae were fixed and stored as controls. Each larva was cut into half along the dorsal line, and both halves were processed for histology (Sakura Tissue-Tek VIP) by embedding in paraffin wax (Thermo Scientific Histostar Embedding Center), and sectioning (4 µm) using a Leica RM2135 before being transferred onto a glass slide, for hematoxylin and eosin (H&E) or Ziehl-Neelsen (ZN) staining. Slides were examined using light microscopy (Nikon Eclipse 80i), and images captured using the NIS-Elements BR imagine software and a Nikon DS-Ri1 camera. For TEM analysis, infected *G. mellonella* larvae (n = 10 per timepoint) at 1, 24, and 168 h post-infection, were bled through needle puncture and their hemolymph pooled into an ice cold 2 ml Eppendorf tube, washed with ice cold PBS, and centrifuged at 800 g for 10 min. The pelleted cells were resuspended in 3% glutaraldehyde (v/v in PBS) and fixed for 10 min, pelleted at 800 g, and stored at 4°C. Subsequently, the cell pellet was post-treated with 1% osmium tetroxide (0.1 M phosphate buffer), dehydrated (in a graded series of alcohol up to 100%), embedded (in epoxy resin, Araldite), cut into 70–90 nm ultra-thin sections, which were mounted onto a grid, stained with 0.5% uranyl acetate (w/v) and 3% lead citrate (w/v), and examined on a Tecnai bioTWIN transmission electron microscope. For confocal microscopy, larvae were infected with SAMTB *lux* (2x10^7^ CFU) and hemolymph were bled through needle puncture at 1 h and 168 h post-infection. Three larvae were bled at each timepoint and pooled (approx. 40 µl/larva) with 700 µl of PBS. The cell suspension was mixed and 200 µl layered onto a 4-well Millicell® EZ SLIDE (Merck, UK) with a further 200 µl of PBS. The cells were incubated at room temperature for 30 min to allow for cell adhesion and washed twice with PBS to removed non-adherent cells. The slides were fixed with 3% formaldehyde for 15 min and washed twice with PBS. SYBR gold (Thermo Fisher, UK) was used to stain SAMTB *lux* bacilli, using the method previously described by Lenaerts and colleagues [19]. SlowFade Gold with 4′,6-diamidino-2-phenylindole (DAPI, Thermo fisher, UK), was used to counterstain the nucleus and mount the slides for imaging on a Leica SP8 inverted confocal microscope.

### Statistical analysis

The experimental data were analyzed using Prism 8 (Graphpad Software Inc, UK). Where appropriate, the non-parametric Kruskal–Wallis test with Dunn’s multiple comparison test or Mann-Whitney test was used.

## Results

### G. mellonella *survival is SAMTB* lux *dose-dependent*

*G. mellonella* larvae (n = 30 per group) were challenged with a range of SAMTB *lux* inocula and survival monitored over 96 h. Survival was dose-dependent, with higher inocula correlating with increased larval mortality (p < 0.0001) (Figure 1). The LD_50_ (dose required to achieve 50% killing) was 2.5×10^7^ CFU. There was no death using PBS-T as the mock infection or with the pricked (simulated injection injury) control group (Figure 1, pricked not shown). SAMTB *lux* virulence is dependent on viability since heat-treated bacteria (2x10^7^ CFU) did not kill larvae (Figure 1). Physiological changes in infected larvae (2x10^7^ CFU) were monitored over 96 h. Infection led to gradual melanization over time, initially manifesting at the dorsal line 48 h post-infection, which became systemic by 168 h post-infection. Additionally, larval motility decreased as the infection progressed. Pupation was observed in larval groups inoculated with PBS, heat-killed and 1×10^7^ CFU SAMTB *lux*. No pupation was observed in larval groups inoculated with 2x10^7^, 5×10^7^ and 1×10^8^ CFU.

### *SAMTB* lux *proliferates in* G. mellonella

**Figure 2.**
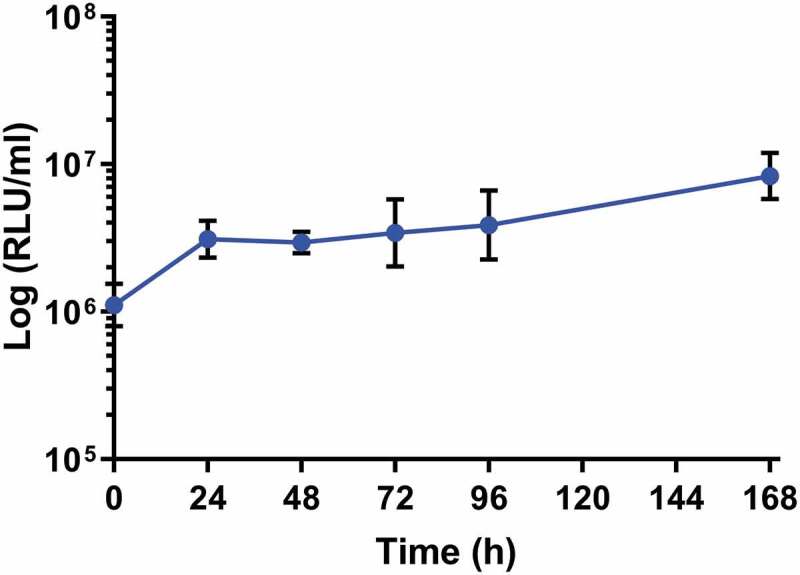
*In vivo* proliferation of SAMTB *lux* in *G. mellonella* larvae over a 168 h time course.

**Figure 3.**
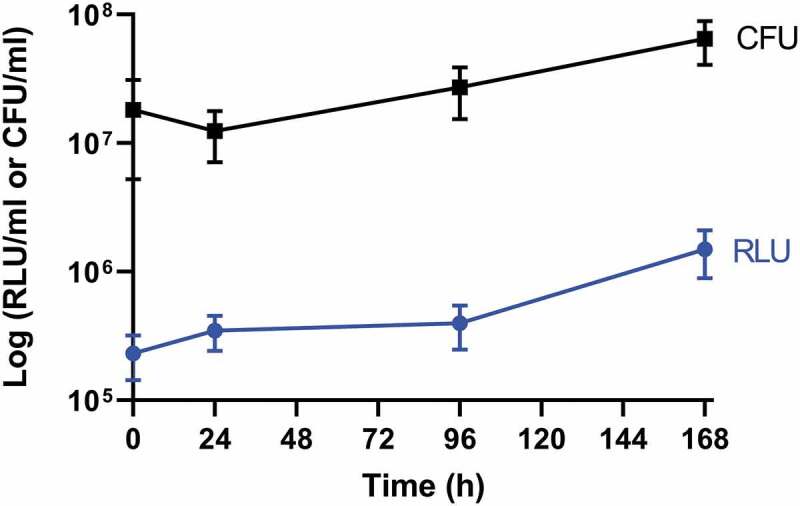
Recovery of SAMTB *lux* from larval homogenate over a 168 h time course.

Larvae were infected with SAMTB *lux* (2x10^7^ CFU) and the changes in *in vivo* load of SAMTB *lux* were monitored over a 168 h time course using bioluminescence (RLU) of the larval homogenates. SAMTB *lux* bioluminescence increased by approximately 0.75 log RLU over 168 h, demonstrating that the bacterium can survive and proliferate within larvae (Figure 2). Post-infection, there was an initial rise in bioluminescence (0.25 log RLU) between 0–24 h; an increase of 0.1 log RLU between 24–96 h; and a 0.4 log RLU increase between 96–168 h. Background bioluminescence of the larval homogenates was low (5000–8000 RLU/ml) with no impact on SAMTB *lux* bioluminescence. The RLU:CFU ratio *in vivo* was determined to be 0.02:1 over a 168 h time course (Figure 3). This ratio was used as the baseline to rapidly quantify relative *in vivo* SAMTB *lux* load. There was a gradual increase, in both the RLU and CFU counts *in vivo* over the 168 h time course, with the exception of the 24 h timepoint, where there was a slight drop in CFU counts relative to the rise in RLU.

*G. mellonella* larvae (n ≤ 30) were infected with 2×10^7^ CFU of SAMTB *lux* and at each timepoint (0, 24, 48, 72, 96 and 168 h), four larvae were individually homogenized, and the bioluminescence (RLU/ml) of the homogenate was measured to quantify the relative abundance of SAMTB *lux in vivo*. Data are pooled from three independent experiments. The means of each group are plotted, and the error bars represent the standard deviation of the mean.

Larvae were infected with 2×10^7^ CFU of SAMTB *lux*. At 0, 24, 96 and 168 h post-infection, four larvae were homogenized and plated onto Middlebrook 7H11 agar containing additional growth supplements (leucine and pantothenate) as well as piperacillin (20 µg/ml) to prevent contamination from larval microbiota. Data are pooled from two independent experiments.

### *Granuloma-like structures develop in* G. mellonella *in response to* *SAMTB* lux *infection*

**Figure 4.**
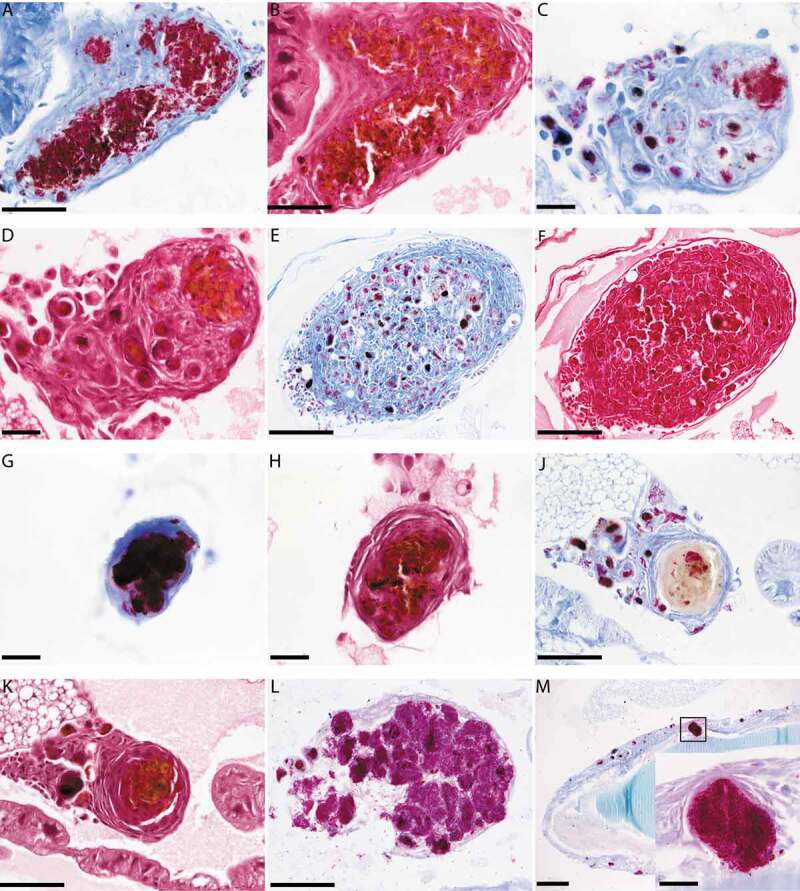
Histological analysis of *G. mellonella*-SAMTB *lux* interaction over time.

**Figure 5.**
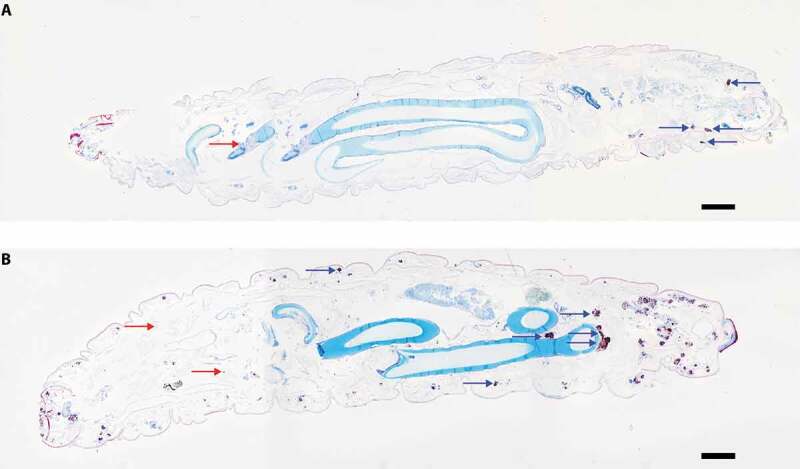
Cross sectional view of whole larvae infected with SAMTB *lux.*

Histological sections from larvae infected with SAMTB *lux* (2x10^7^ CFU) were stained with H&E or ZN to visualize host-SAMTB *lux* interaction. As early as 24 h post-infection, acid fast bacilli were organized in foci that were surrounded by host cells to form granuloma-like structures (Figure 4a-c). In these structures individual bacilli formed foci of variable bacterial density, with some granuloma-like structures containing compact clusters of acid fast amorphous material. H&E staining of the same section correlated to these structures as deep red/dark brown or black color formations for compact acid fast structures, and golden brown for areas containing high numbers of individual bacilli. Dispersed individual bacilli were not detectable in H&E sections (Figure 4b-d). The morphology of the granuloma-like structures varied considerably throughout all stages of infection with a mixture of individually identifiable bacilli and dense bodies with high affinity for ZN staining (Figure 4e–f), and discreet dense material composed almost exclusively of acid fast material (Figure 4g–h). Some of these densely packed structures at 168 h post-infection lost their affinity for the ZN stain (Figure 4j), although they remained compact (Figure 4k). Dissemination of prolific SAMTB *lux* bacilli were visualized at 168 h post-infection (Figure 4l). Occasionally bacilli appeared to migrate from the hemolymph to the internal organs (Figure 4m); within the resolution limits of light microscopy no host reaction was evident. In ZN labeled sections individual bacilli, not associated with host cells, were also identified within the hemolymph at all stages of the infection. As the infection progressed from 24 to 168 h, granuloma-like structures containing SAMTB *lux* bacilli were more numerous and found present throughout the body of the larvae (Figure 5a,b).

Histological sections of *G. mellonella* infected with SAMTB *lux* stained with Ziehl-Neelsen (ZN) or hematoxylin and eosin (H&E). (A) At 24 h post infection, large numbers of individual and densely organized bacilli were stained with ZN. (B) The H&E section of the same granuloma-like structure shows host cells occupying spaces which, in the ZN section, are filled with bacilli. Loss of eosinophilia from the H&E section gives rise to a brown pigmentation which co-localizes with clumping of bacilli in the ZN section. (C) As early as 24 h post-infection, granuloma-like structures may contain independent colonies of bacilli and compact ZN reactive amorphous material. (D) The H&E section of the same structure shown in C; loss of eosinophilia coincides with the existence of a large colony of bacilli. (E-F) At 168 h post-infection, granuloma-like structures containing compact ZN positive substances and individual bacilli or colonies of bacilli, were observed. At 48 h post-infection, (G) ZN and (H) H&E staining, show a single compact granuloma with a ZN reactive core. Such granuloma-like structures can be observed as single entities or can be included in formations that include multiple compact ZN positive structures similar to panels C-F. (J-K) Granuloma-like structure at 168 h post-infection, stained with ZN and H&E respectively, shows a single compact granuloma structure. However, the ZN affiliation of the amorphous central core has been abolished to a large extend implying breakdown of the structures that bind to the ZN dye. (L) Swarms of ZN positive bacilli form a granuloma-like structure. Part of the granuloma wall is impaired allowing access of bacteria to the surrounding hemolymph. (M) shows bacilli invading and forming colonies in internal organs of a larva. There is no evident host reaction to invading bacilli. The inset shows a larger magnification of the rectangle enclosed structure which shows abundant proliferation of bacilli. Scale bar in A, B, J, K and L represents 50 µm; in C, D, G, H and the M inset, scale bar represents 20 µm; while in E, F and M the scale bar represents 100 µm.

Histological sections of whole *G. mellonella* larvae infected with SAMTB *lux* 24 h (A) and 168 h (B) post challenge showing multiple granuloma-like structures. Blue arrows indicate formations where there is an abundance of active bacilli which may be also accompanied by more consolidated structures similar to those presented in Figure4 (a,c,e,l). Red arrows show formations that are similar to those presented in Figure 4(g). Scale bar represents 1000 µm.

### *Innate immune cells of* G. mellonella *rapidly internalize SAMTB* lux *following infection*

**Figure 6.**
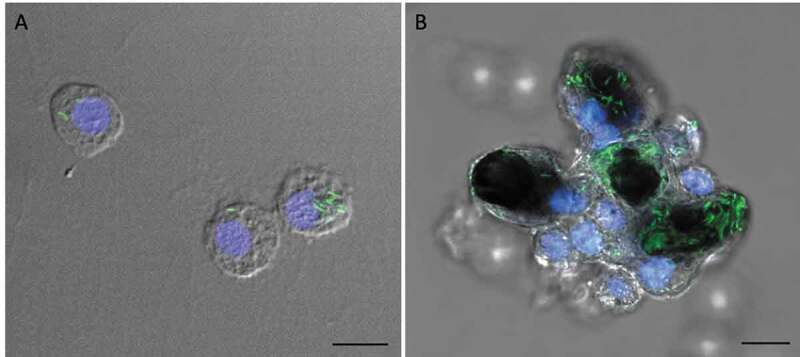
SAMTB *lux* associates with hemocytes following infection.

**Figure 7.**
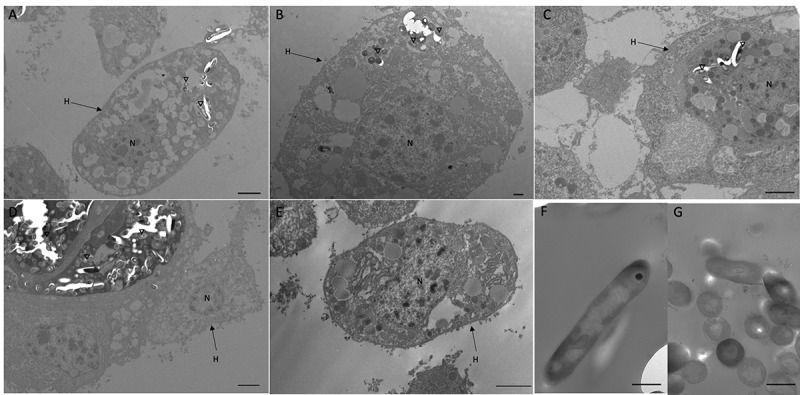
Interaction between SAMTB *lux* and phagocytic hemocytes.

Confocal microscopy and TEM was carried out on hemolymph from infected larvae to visualize hemocyte–SAMTB *lux* interaction. Confocal microscopy visualized the association of SAMTB *lux* bacilli with hemocytes at 1 h post-infection (Figure 6a) and 168 h post-infection (Figure 6b), with the later timepoint showing a nodule like structure containing a number of central SAMTB *lux* clusters surrounded by hemocytes. TEM further confirmed that SAMTB *lux* had been internalized by hemocytes as early as 1 h post-infection (Figure 7a,b). There is clear evidence of early phagocytosis associated with interaction and formation of membrane invaginations. SAMTB are also clearly internalized at all timepoints up to and including 168 h post-infection (Figure 7c,d). Structural references of SAMTB *lux* alone, in different geometric orientations, are provided in Figure 7(f,g). TEM confirmed the histopathological findings, suggesting that SAMTB *lux* invades and/or is phagocytosed by hemocytes and multiplies within to form aggregate-like structures.

Confocal microscopy was carried out on hemocytes extracted from *G. mellonella* larvae infected with SAMTB *lux* (2x10^7^ CFU) at (a) 1 h and (b) 168 h post-infection. Early association between hemocyte and SAMTB *lux* was seen at 1 h post-infection (a). Aggregates of hemocytes in an early granuloma like structure surrounding multiple clusters of SAMTB *lux* was observed at 168 h post-infection (b). These images are a superimposition of DAPI (nucleus, blue), SYBR gold (SAMTB *lux*, green) and differential interference contrast (DIC) image. Scale bars represents 10 µm.

Transmission electron microscopy (TEM) was carried out on hemocytes extracted from *G. mellonella* larvae infected with SAMTB *lux* (2x10^7^ CFU) at: (a, b) 1 h (c) 24 h and (d) 168 h post infection. (e) shows healthy non-infected hemocytes. (f, g) show SAMTB *lux* bacilli in various geometric orientations. Internalization of SAMTB *lux* was observed as early as 1 h post-infection and presence of the bacilli persisted 24 h post-infection (A-C). By 168 h post-infection, aggregates of SAMTB *lux* bacilli, surrounded by hemocytes in an early granuloma-like structure, could be observed under TEM (D). H = hemocytes, N = nucleus, inverted triangles indicate SAMTB *lux*. Scale bars represent (a, c-e): 2 µm, (b, f, g): 500 nm.

### *SAMTB* lux *infection induce a transient increase in circulating hemocyte counts*

**Figure 8.**
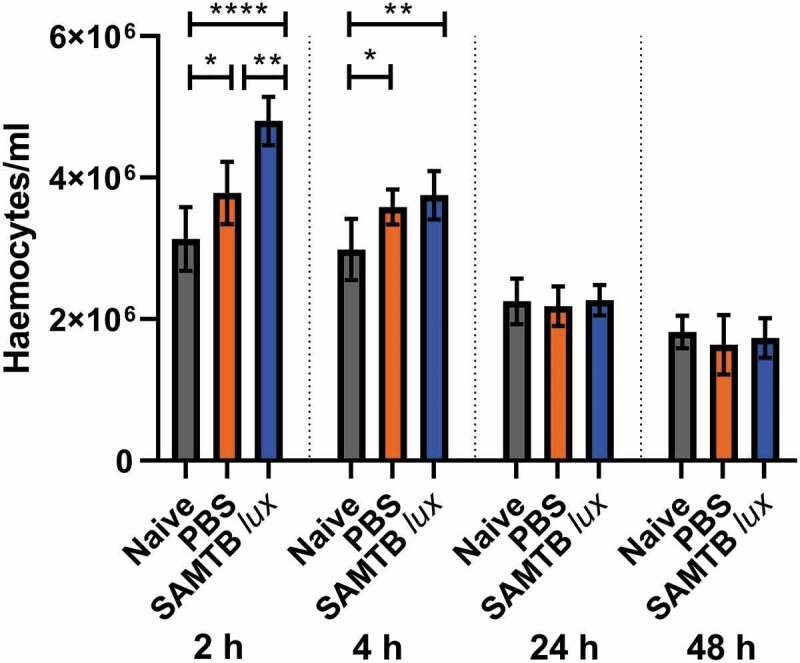
Changes in hemocyte counts in SAMTB *lux* infected larvae.

A significant increase in *G. mellonella* hemocyte counts was found at both 2 and 4 h for both the PBS injected larvae (p < 0.05 and p < 0.05 each timepoint, respectively) and SAMTB *lux* infected larvae (p < 0.0001 and p < 0.01, respectively for each timepoint) when compared to naïve larvae (Figure 8). However, hemocyte counts rapidly normalized to the level of naïve larvae by 24 h for both groups. Additionally, hemocyte counts were significantly higher in SAMTB *lux* infected compared to PBS treated larvae at 2 h (p < 0.01). However, by 4 h there was no significant difference in hemocyte counts between the two groups.

Total number of circulating hemocytes of naïve, PBS-T injected and SAMTB *lux* infected (2x10^7^ CFU) larvae were determined at 2, 4, 24 and 48 h post-injection. Five larvae were bled (approximately 60 µl of hemolymph/larva) and diluted in PBS containing 0.37% of mercaptoethanol. Hemocytes were stained 1:1 with trypan blue (0.4%) and counted in a hemocytometer. Data are pooled from three independent experiments, each with two technical repeats. Plotted are the means of each group and the error bars represent the standard deviation of the mean. PBS and SAMTB *lux* infected larvae were compared to the naïve control using one-way ANOVA with Dunnett’s multiple comparison test. * P < 0.05, ** P < 0.01, *** P < 0.001, **** P < 0.0001.

### *The* G. mellonella *– SAMTB* lux *infection model can be used to screen for drug efficacy*

**Figure 9.**
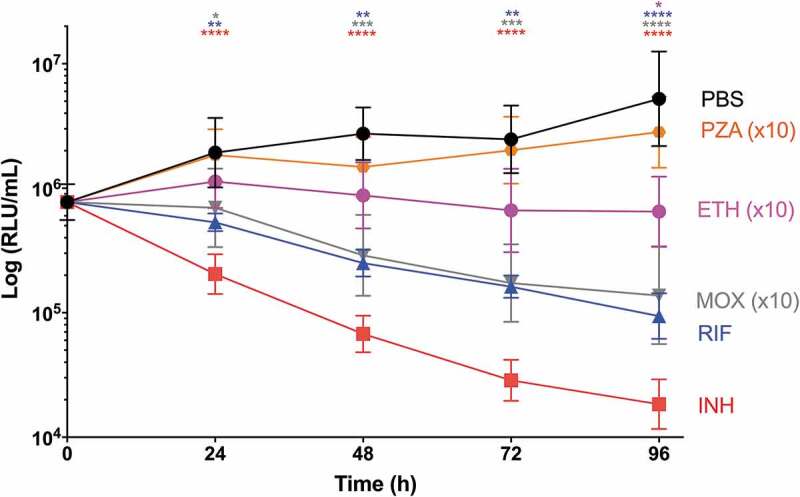
The effect of first-line and second-line antibiotics on the reduction of SAMTB *lux* bioluminescence within *G. mellonella* over a 96 h time course.

**Figure 10.**
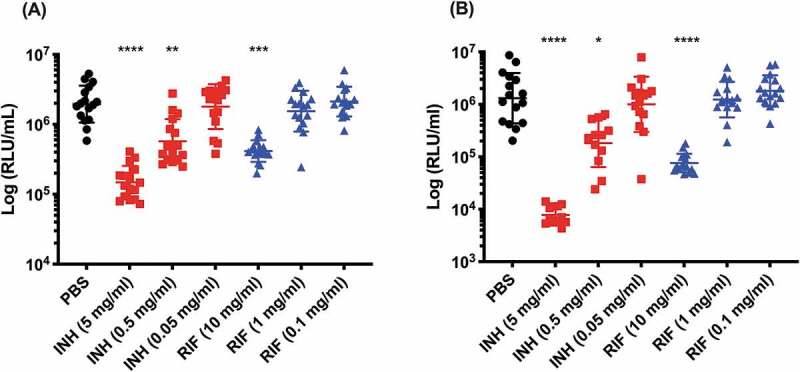
The effect of INH and RIF concentration on the reduction of SAMTB *lux* bioluminescence within *G. mellonella.*

**Figure 11.**
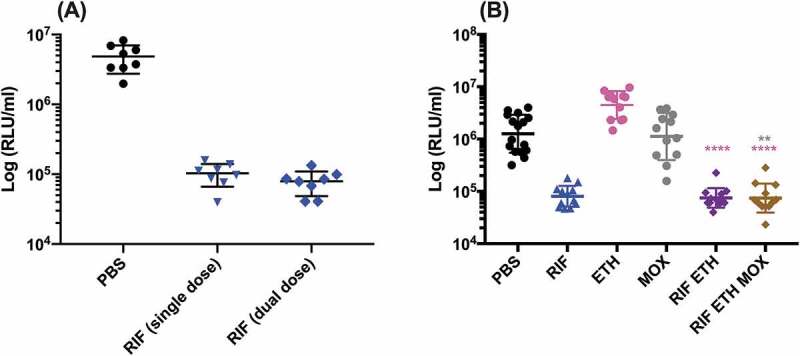
The effect of RIF multiple dosing and RIF combination drug therapy on the reduction of SAMTB *lux* bioluminescence within *G. mellonella.*

SAMTB *lux* infected *G. mellonella* larvae were treated with first-line, INH, RIF, PZA and ETH; and second-line, MOX and BDQ antibiotics at recommended adult clinical dosage relative to larval weight, to evaluate the use of this *in vivo* model as a drug screen. The *in vitro* MIC of the antibiotics against SAMTB *lux* were: INH (0.25 µg/ml), RIF (< 0.125 µg/ml), BDQ (< 0.125 µg/ml), ETH (0.5 µg/ml), PZA (> 16 µg/ml), and MOX (0.25 µg/ml). Drug efficacy was determined by comparing the change in the *in vivo* bioluminescence of SAMTB *lux* between the treated and mock treated (PBS-T) control over a 96 h time course. Treatment led to significant reductions in SAMTB *lux* bioluminescence for INH (P < 0.0001) and RIF (p < 0.01) treated larvae, but not for BDQ, ETH, MOX and PZA 96 h post-treatment at the recommended adult clinical dose relative to the larvae body weight (Figure 9, Supplementary Figure 2). When treated with a ten-fold higher dose of ETH, MOX or PZA, a significant reduction in SAMTB *lux* bioluminescence was observed for MOX (x10) at all timepoints (with P < 0.0001 by 96 h post-infection); while ETH (x10) showed a trend in the reduction of SAMTB *lux* bioluminescence, with significant reduction at 96 h post-treatment (p < 0.05) when compared to the control (Figure 9). No significant changes in RLU were detected in PZA (x10) treated larvae when compared to the control. Due to their substantial bioluminescence reducing activity on SAMTB *lux*, INH and RIF were further screened at three different doses, which demonstrated a clear dose-dependent effect (Figure 10). The effects of single and dual dosing for RIF was tested. INH was not used in multiple drug combination studies as any further improvements in drug efficacy, and hence reduced bioluminescence, were likely to result in RLU measurements indistinguishable from background bioluminescence of larval homogenates (~5000 RLU). Dual RIF treatment did not improve drug efficacy over single dose treatment, as both single and dual dosing achieved similar levels of drug efficacy over the 96 h time course (Figure 11a). Furthermore, the use of RIF in combination with ETH and/or MOX, (both of which did not show any significant antimycobacterial activity in this model against SAMTB *lux*) at clinically recommended dosages for adult TB treatment relative to larval body mass (Supplementary Figure 2), was tested with RIF as a dual (RIF/ETH) and triple (RIF/ETH/MOX) combination treatment. Both dual and triple combination treatments resulted in improved drug efficacy over the use of either ETH or MOX alone (Figure 11b). However, no improvement was seen over the use of RIF alone.

Larvae (n = 30/group) were infected with 2×10^7^ CFU of SAMTB *lux*. At 1 h post-infection, larvae were treated with a single dose of INH (5 mg/kg), RIF (10 mg/kg), and BDQ (5.7 mg/kg) at the recommended clinical dosage relative to the larval body weigh; while PZA (250 mg/kg), ETH (150 mg/kg), MOX (67 mg/kg) were used at concentrations ten-folds higher than the recommended dosage. Control groups were injected larvae mock treated with PBS-T. At 0, 24, 48, 72, and 96 h post-infection, four larvae from each group were individually homogenized and the bioluminescence (RLU/ml) of the homogenates was measured to determine the relative drug efficacies. Data are pooled from three independent experiments. Plotted are the mean of each group and the error bars represent the standard deviation of the mean. Non-parametric Kruskal-Wallis test with Dunn’s multiple comparison was carried out against the PBS-T injected control for all treatment groups. * P < 0.05, ** P < 0.01, *** P < 0.001, **** P < 0.0001.

Larvae (n = 30/group) were infected with 2 × 10^7^ CFU of SAMTB *lux*. At 1 h post-infection, larvae were treated with a single dose of INH (5 mg/kg) or RIF (10 mg/kg). Control groups were injected larvae mock treated with PBS-T. At (A) 24 h and (B) 96 h post-infection, four larvae from each group were individually homogenized and the bioluminescence (RLU/ml) of the homogenate was measured to determine relative drug efficacy. Data are pooled from two independent experiments. Plotted are the mean for each group and the error bars represent the standard deviation of the mean. Non-parametric Kruskal-Wallis test with Dunn’s multiple comparison was carried out against the PBS-T control for all treatment groups. * P < 0.05, ** P < 0.01, *** P < 0.001, **** P < 0.0001.

Larvae (n = 30/group) were infected with 2×10^7^ CFU of SAMTB *lux*. (a) At 1 h post-infection, larvae were treated with a single dose of RIF (10 mg/kg) and for dual dosing an additional dose was given 24 h post-infection. (b) At 1 h post-infection, larvae were treated with a single dose of RIF (10 mg/kg), ETH (15 mg/kg), MOX (6.7 mg/kg) or in combination as RIF/ETH or RIF/ETH/MOX. At 96 h post-infection, four larvae from each group were individually homogenized and the bioluminescence (RLU/ml) of the homogenate was measured to determine relative drug efficacy. The efficacy of dual RIF dosing was compared to single dosing using the Mann-Whitney test. As for combination drug therapy, the efficacy of each drug combination was compared to their respective single drug type efficacy using a non-parametric Kruskal-Wallis test with Dunn’s multiple comparison. For combination drug therapy, the color of * correlates to the color of the single drug type. * P < 0.05, ** P < 0.01, *** P < 0.001, **** P < 0.0001.

## Discussion

We sought to determine whether *G. mellonella* could be used as a surrogate host for SAMTB *lux* thereby allowing experiments to be performed at BSL2. Virulence of SAMTB *lux* in *G. mellonella* was dependent on viability and inoculum size. The LD_50_ dose of SAMTB *lux* (2.5x10^7^ CFU) was of a similar order of magnitude to that of BCG (1x10^7^ CFU) [15]. In immunocompromised SCID mice SAMTB was considered attenuated compared to BCG or wild-type MTB H37Rv, since BCG-infected mice had lower survival outcome compared to those challenged with SAMTB [20]. In *G. mellonella*, SAMTB *lux*, unlike BCG [15], proliferated. Our results are in contrast with those in guinea pigs and mice where SAMTB or the single auxotrophic MTB mutant (Δ*leuD*) did not replicate [20,21]. We speculate that SAMTB was unable to replicate as there was insufficient availability of leucine and pantothenate in these hosts to overcome the auxotrophy. In *G. mellonella*, leucine comprised 4.91% of the total metabolome detected [22], and common insect feed for rearing *G. mellonella* typically contains 10.8 g/kg of leucine and 32.8 mg/kg of pantothenic acid [23]. While larvae were not fed during the experiments, we speculate that the residual abundance of leucine and pantothenate allowed auxotrophic SAMTB *lux* to proliferate *in vivo*. Furthermore, the abundance of these amino acids, likely contributed to the initial increase RLU 24 h post-infection as SAMTB *lux* metabolism adapted to this nutrient rich environment.

Histological analysis identified that the interaction between *G. mellonella* larvae and SAMTB *lux* was similar to that of BCG *lux* [15]. However, unlike BCG infection where most bacilli were contained and eventually digested into acid fast amorphous masses by the innate immune system, some SAMTB *lux* bacilli appear to replicate uninhibited within smaller granuloma-like foci, with such structures increasing in number and size over time. Furthermore, clusters of SAMTB *lux* bacilli were found at 168 h, suggesting that the initial containment within granuloma-like structures was overcome by rapidly multiplying bacteria. These observations agree with *in vivo* proliferation of SAMTB *lux* between 96 and 168 h post-infection. While the cellular composition of *G. mellonella* granuloma-like structures is unknown, it is likely that they contain a mixture of hemocyte sub-types: plasmatocytes, granular cells, and oenocytoids. These hemocytes are integral components of phagocytosis, nodulation (co-ordinated adherence or internalization of bacteria), and melanization [24]. Confocal microscopy clearly showed an association of SAMTB *lux* bacilli with hemocytes at 1 h post-infection, which developed into nodules resembling early granuloma-like structures at 168 h post-infection. As images lacked cytoskeletal staining, the geometric location of the bacilli in relationship to the hemocyte could not be identified. However, TEM visualized rapid internalization of SAMTB *lux* 1 h post-infection, which is in line with prior reports of phagocytic activity against non-tuberculous mycobacteria (NTM) [25], and the SAMTB *lux*-hemocyte association seen from the confocal microscopy images. Presence of internalized SAMTB *lux* bacilli within hemocytes were observed throughout the 168 h time course with aggregation of internal bacilli, similar to those observed in the BCG *lux* infection [15] and that of the confocal microscopy image seen at the same timepoint. However, unlike BCG *lux* infection, SAMTB *lux* bacilli did not accumulate lipid bodies with progression of the infection, likely reflecting the proliferative nature of SAMTB *lux* in comparison to BCG *lux*. Future studies will characterize the interaction between the hemocytes and MTB, including cellular signals (e.g. apoptosis/necrosis) or lysosomal activity following infection.

Characterization of the *G. mellonella* innate immune response revealed that infection with SAMTB *lux* resulted in a temporary increase in circulating hemocyte numbers at 2 and 4 h post-infection, which normalized to the basal level of naïve larvae by 24 h. Larval response to mycobacterial infection were previously characterized by Entwistle and Coote [25] using NTM (*M. marinum* and *M. fortuitum*) at a similarly lethal dose. Unlike our study, NTM infection led to an increase in circulating hemocyte numbers throughout the 48 h time course, when compared to the naïve and PBS controls [25]. The transient rise in our study is likely a basal response to fluid injection, rather than a specific response to SAMTB *lux*, as no significant difference between the PBS injected and SAMTB *lux*-infected control groups was identified 4 h post-infection. Infection with fast-growing bacterial pathogens, e.g. *Legionella pneumophila* and *Staphylococcus aureus*, led to 3–6 fold increases in hemocyte counts [26,27]. We speculate that a greater rate of bacterial replication may be associated with an increase in circulating hemocyte counts.

Treatment of SAMTB *lux* infected larvae with INH and RIF resulted in rapid reduction in SAMTB *lux* bioluminescence. This was unsurprising as both drugs are highly effective antimycobacterials *in vitro* and *in vivo* [28,29]. Despite *in vitro* activity, BDQ, ETH and MOX did not result in a significant reduction in SAMTB *lux* bioluminescence at the standard dosage. Similar observations were made for larvae infected with BCG, where treatment with ETH and MOX or PZA had no efficacy [13]. However, the use of ETH or MOX at a ten-fold higher concentration than that of the clinically recommended dosage, resulted in significant reduction in SAMTB *lux* bioluminescence. This induction of drug efficacy *in vivo* is a clear indication that this model is sensitive to drug dosage, underlining the importance of testing a range of drug doses. This is important as our knowledge of the pharmacokinetic (PK) and pharmacodynamic (PD) properties of antibiotics in *G. mellonella* is currently limited [30]. PZA was inactive *in vitro* and *in vivo* at both standard and ten-fold higher concentrations. PZA was expected to be inactive against SAMTB *lux* as it lacks *panD* which synthesizes the enzyme aspartate decarboxylase, a known target of pyrazinoic acid [31]: this is evident from the lack of efficacy when used at a ten-fold higher concentration to that of the standard dosage. Repeated dosing of RIF alone or in combination with ETH, or ETH and MOX at the standard dosage, did not reduce SAMTB *lux* bioluminescence any further than RIF alone. Combination drug therapy is the gold standard for the treatment of TB. However, the primary use of a cocktail of antimycobacterials is to minimize the development of drug resistant TB during the lengthy treatment period and not for synergistic activities [18]. Further PK/PD research is required to maximize the use of *G. mellonella* in screening for novel antibiotics.

The use of RLU as a read-out of mycobacterial viability within *G. mellonella* is advantageous and allows for the rapid evaluation of drug efficacy, acting as a pre-screen for candidate compounds prior to mammalian testing. Such application would greatly increase the number of compounds that could be tested at a fraction of the cost and time of conventional *in vivo* studies. Furthermore, pre-screening would significantly reduce the number of mammals that are often used as antimicrobial activities do not always correlate between *in vitro* and *in vivo* conditions. However, it is important to note that RLU alone cannot determine the mechanism behind the antimycobacterial activity (i.e. bactericidal/bacteriostatic effects), as RLU production is a measure of the metabolic activity of SAMTB *lux* [32]. Therefore, RLU simply serves as a rapid measure of drug efficacy, that does not rely on time consuming CFU enumeration. A more comprehensive study into the antimycobacterial activity of candidate compounds is essential during mammalian studies.

*D. rerio* is a popular non-mammalian TB infection model, as it is genetically tractable, high throughput, financially sustainable, and is ethically more acceptable than conventional mammalian counterparts (with the exception of adult zebrafish) [33]. Limitations of *D. rerio* include the use of *M. marinum* due to biological incompatibility (cannot be incubated at 37°C) [6,34], and the need for specialized rearing facilities and regular maintenance [35]. In contrast, *G. mellonella* can be incubated at 37°C and requires no specialized facility or maintenance, making the study of the MTBC easier and more accessible to the wider research community. Incubation temperature is a key factor as this can affect mycobacterial physiology [36], and the ability to study virulence at human temperatures is highly beneficial. A current limitation of *G. mellonella* is the lack of a fully annotated genome. However, an extensive transcriptomic database [37], and an unannotated genome [38] are available. A fully annotated genome and the development of methods to genetically manipulate *G. mellonella*, as described for other Lepidoptera [39], will facilitate uptake of the BSL2-compliant SAMTB *lux* infection model.

In conclusion, this study demonstrates the use of *G. mellonella* as an infection model for MTB, which lacks both the conventional ethical limitations associated with the use of mammalian infection models, and the need for BSL3 facilities. Infection of *G. mellonella* with SAMTB *lux* led to the proliferation of the bacilli *in vivo* and the formation of granuloma-like structures. Furthermore, this model can be used as a drug screen for antimycobacterial agents. Further optimization and characterization of the model will be carried out in the future, with specific focus on the proteomic, metabolomic and cellular response to infection. The successful uptake of this model in the MTB research community could significantly increase research throughput, while considerably reducing the number of mammalian models used in research.

## Supplementary Material

Supplemental Material

Supplemental Material
