# Supplementary material for: A novel biosafety level 2 compliant tuberculosis infection model using a ΔleuDΔpanCD double auxotroph of Mycobacterium tuberculosis H37Rv and Galleria mellonella
Source: Virulence. 2020 Jun 24;11(1):811–24. doi: 10.1080/21505594.2020.1781486 (PMC7550006; doi:10.1080/21505594.2020.1781486)
Supplement: Supplemental Material [file KVIR_A_1781486_SM2316.docx]

**
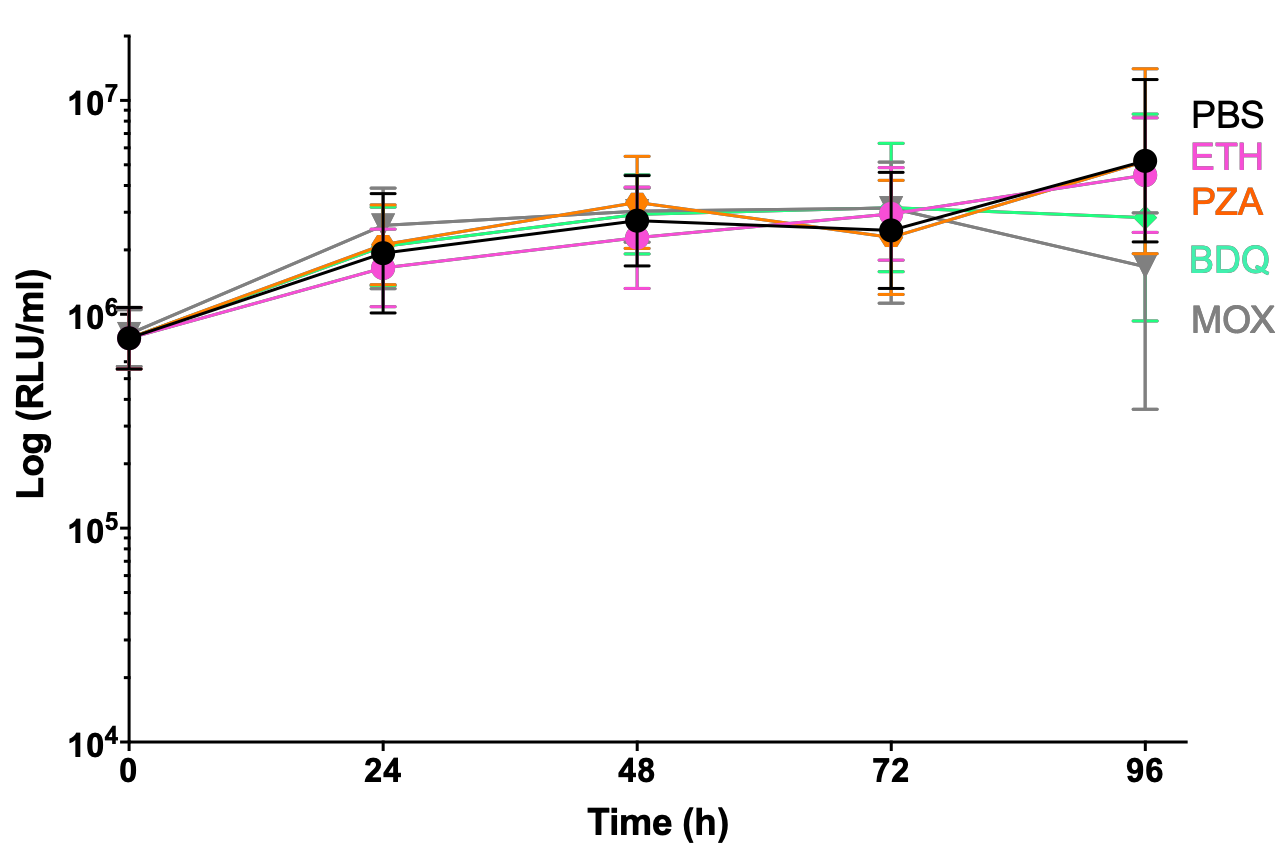
**

**Supplementary Figure 2. The effect of first-line and second-line antibiotics on the reduction of SAMTB *lux* bioluminescence within *G. mellonella* over a 96 h time-course.** Larvae (n=30/group) were infected with 2x10^7^ CFU of SAMTB *lux*. At 1 h post-infection, larvae were treated with a single dose of PZA (25 mg/kg), ETH (15 mg/kg), MOX (6.7 mg/kg), BDQ (5.7 mg/kg). Control groups were injected larvae mock treated with PBS-T. At 0, 24, 48, 72, and 96 h post-infection, four larvae from each group were individually homogenised and the bioluminescence (RLU/ml) of the homogenates was measured to determine the relative drug efficacies. Data are pooled from three independent experiments. Plotted are the mean of each group and the error bars represent the standard deviation of the mean. Non-parametric Kruskal-Wallis test with Dunn’s multiple comparison was carried out against the PBS-T injected control for all treatment groups. * P < 0.05, ** P < 0.01, *** P < 0.001, **** P < 0.0001.
