# Supplementary material for: A novel biosafety level 2 compliant tuberculosis infection model using a ΔleuDΔpanCD double auxotroph of Mycobacterium tuberculosis H37Rv and Galleria mellonella
Source: Virulence. 2020 Jun 24;11(1):811–24. doi: 10.1080/21505594.2020.1781486 (PMC7550006; doi:10.1080/21505594.2020.1781486)
Supplement: Supplemental Material [file KVIR_A_1781486_SM2113.docx]

**
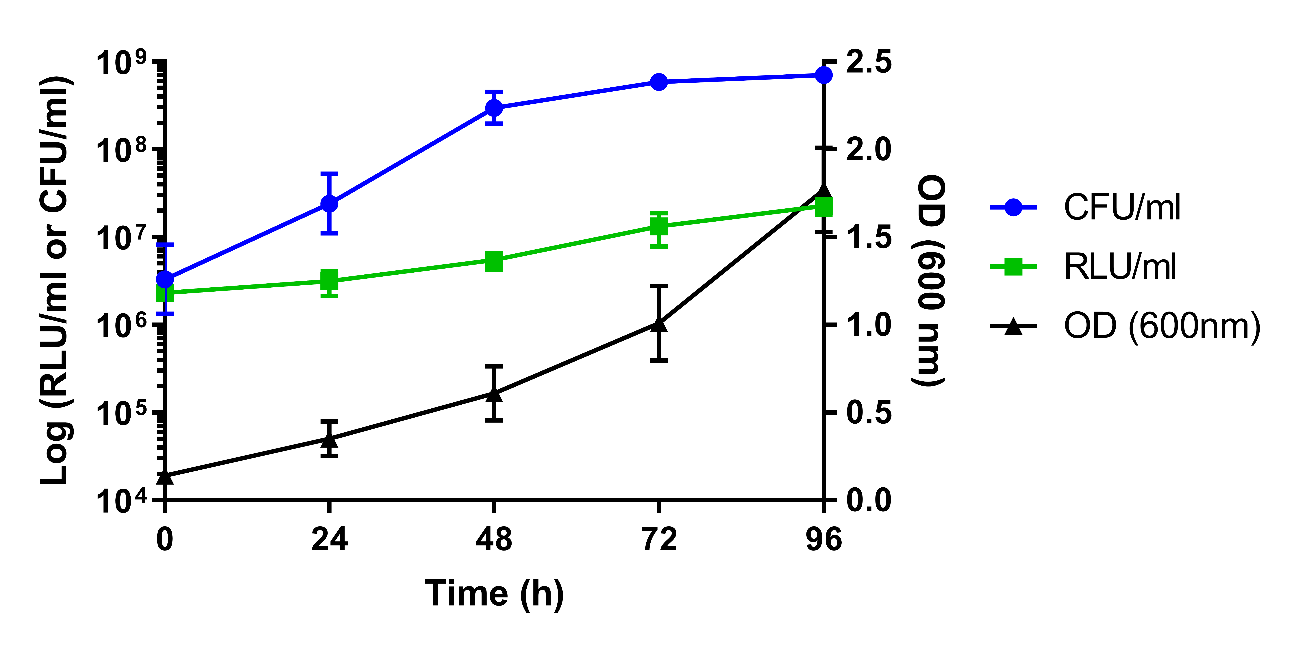
**

**Supplementary figure 1. *In vitro* growth curve of SAMTB *lux*.** The growth of SAMTB *lux* in Middlebrook 7H9 media, supplemented with leucine and pantothenate was measured using bioluminescence (RLU), CFU, and OD (600nm) over a period of 96 h with time-points every 24 h. Results represent a minimum of two independent experiments.
